# A new protocol for the synthesis of 4,7,12,15-tetrachloro[2.2]paracyclophane

**DOI:** 10.3762/bjoc.12.237

**Published:** 2016-11-17

**Authors:** Donghui Pan, Yanbin Wang, Guomin Xiao

**Affiliations:** 1 School of Chemistry and Chemical Engineering, Southeast University, 2 Dongnan Daxue Road, Nanjing, Jiangsu, 211189, P. R. China

**Keywords:** bromination, dimerization, H_2_O_2_–HBr system, paracyclophane, polymerization inhibitor

## Abstract

We report a green and convenient protocol to prepare 4,7,12,15-tetrachloro[2.2]paracyclophane, the precursor of parylene D, from 2,5-dichloro-*p*-xylene. In the first bromination step, with H_2_O_2_–HBr as a bromide source, this procedure becomes organic-waste-free and organic-solvent-free and can appropriately replace the existing bromination methods. The Winberg elimination–dimerization step, using aqueous sodium hydroxide solution instead of silver oxide for anion exchange, results in a significant improvement in product yield. Furthermore, four substituted [2.2]paracyclophanes were also prepared in this convenient way.

## Introduction

**Figure 1:**
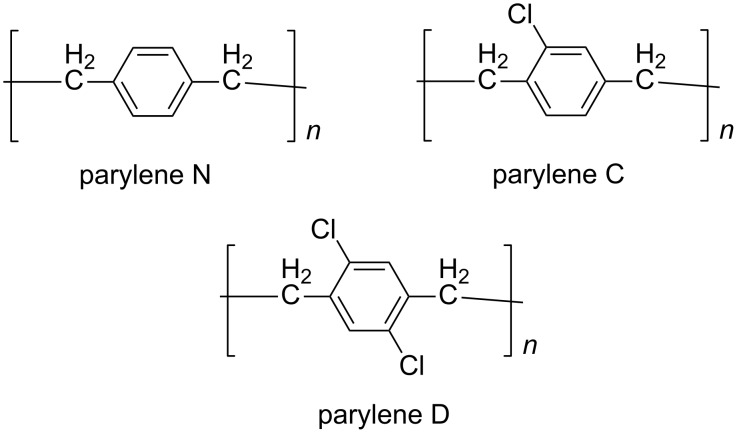
Chemical structures of parylene N, parylene C, and parylene D.

**Figure 2:**
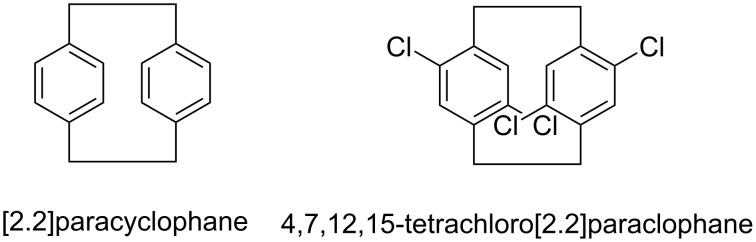
Chemical structures of [2.2]paracyclophane and 4,7,12,15-tetrachloro[2.2]paracyclophane.

Parylene films (Figure 1) are desired uniform coating materials that are widely used in microelectronic engineering, automotive and medical industries, owing to their low dielectricity, high thermal and oxidative stability, and chemical inertness [1–4]. Parylene N was firstly commercialized, and its precursor [2.2]paracyclophane (Figure 2) was typically produced by Hofmann elimination [5–6]. As reported, the uniform coating properties of parylene films were improved by introducing halogen atoms to the structure of the parent [2.2]paracyclophane [7]. Therefore, the two chloride atoms on the benzene ring make parylene D superior to parylene N and parylene C. There are some creative strategies for the synthesis of 4,7,12,15-tetrachloro[2.2]paracyclophane (Figure 2), the precursor of parylene D [8]. Theoretically, direct chlorination of [2.2]paracyclophane is an ideal route to prepare tetrachloroparacyclophane, but a pure polysubstituted product is difficult to obtain by electrophilic substitution without repeated crystallization or chromatographic purification [9]. Thus, we report an improved synthesis method using the Winberg dimerization of 2,5-dichloro-(4-methylbenzyl)trimethylammonium hydroxide without tedious purification.

The important chemical 1-(bromomethyl)-2,5-dichloro-4-methylbenzene is an intermediate in the preparation of 2,5-dichloro-(4-methylbenzyl)trimethylammonium hydroxide. During our investigation of the synthesis of 4,7,12,15-tetrachloro[2.2]paracyclophane, we also adopted an improved bromination process to prepare 1-(bromomethyl)-2,5-dichloro-4-methylbenzene. Traditionally, there are several disadvantages when molecular bromine is used as a brominating reagent, such as toxicity, inconvenient handling and high reactivity, which lead to unsatisfactory results in the bromination process [10–12]. In addition, the release of corrosive HBr as a byproduct and the use of organic solvents make this protocol less environmentally friendly [13]. The use of other brominating agents, such as *N*-bromosuccinimide (NBS) and pyridinium tribromides, also has the drawbacks such as low atom efficiency and the requirement of reagent residue elimination [14]. In contrast to traditional brominating reagents, the H_2_O_2_–HBr system, which generates active bromine in situ, is a convenient and green brominating agent [15]. Furthermore, the use of the H_2_O_2_–HBr couple improves the selectivity and allows for the complete utilization of bromine atoms, thus increasing the atom economy [16]. These advantages prompted us to develop a novel method to prepare 1-(bromomethyl)-2,5-dichloro-4-methylbenzene and 4,7,12,15-tetrachloro[2.2]paracyclophane in a convenient and green way.

## Results and Discussion

We initially planned to optimize the reaction conditions for the bromination of the benzylic position of 2,5-dichloro-*p*-xylene (**1**) by using the H_2_O_2_–HBr system, and investigated various factors, including the activation mode, the reagent stoichiometry, the solvent, and the reaction temperature (Table 1).

**Table 1:** Bromination of 2,5-dichloro-*p*-xylene (**1**) with H_2_O_2_–HBr.

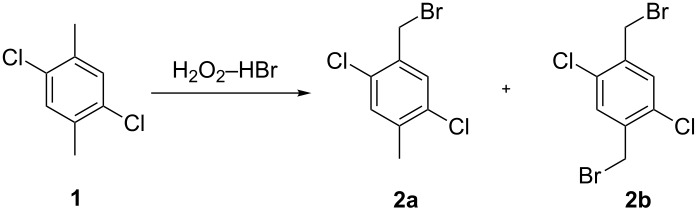							

Entry	**1**/H_2_O_2_/HBr	Mode of initiation^a^	Solvent	Method^b^	Temp. (°C)	Yield^c^ (%)	

						**2a**	**2b**

1	1:1:1	dark	CCl_4_	A	25	22.8	–
2	1:1:1	dark	CCl_4_	A	75	62.9	4.2
3	1:1:1	3% DBP	CCl_4_	A	75	65.8	8.1
4	1:1:1	3% AMPA	CCl_4_	A	75	62.3	7.8
5	1:1:1	incandescent light	CCl_4_	A	25	70.2	3.5
6	1:1:1	incandescent light	H_2_O	A	25	68.8	2.5
7	1:1.5:1	incandescent light	H_2_O	A	25	73.1	4.6
8	1:2:1	incandescent light	H_2_O	A	25	80.4	4.2
9	1:2:1.1	incandescent light	H_2_O	A	25	85.1	3.5
10	1:2:1.5	incandescent light	H_2_O	A	25	82.7	10.1
11	1:2:1.1	incandescent light	H_2_O	B	25	89.9	1.2
12	1:2:1.1	incandescent light	H_2_O	B	80	65.1	28.2

^a^Radical initiators: DBP (dibenzoyl peroxide), AMPA (2,2’-azobis(2-methylpropionamidine) dihydrochloride), 40 W incandescent light bulb. ^b^Method A: H_2_O_2_ and HBr were added in one portion; Method B: H_2_O_2_ was added gradually (1 equiv per 2.5 h). ^c^Yields were determined by ^1^H NMR spectroscopy and were based on starting compound **1**.

The bromination reaction activated by heating in the dark produced a 62.9% yield of the monobrominated product 1-(bromomethyl)-2,5-dichloro-4-methylbenzene (**2a**) accompanied by a small amount of 1,4-bis(bromomethyl)-2,5-dichlorobenzene (**2b**) (Table 1, entry 2). Next, a radical reaction was induced by adding 3 mol % of radical initiator (DBP or AMPA) and proceeded at 75 °C for 4 h (Table 1, entries 3 and 4). Though the yields in both processes increased, the selectivity of **2a** decreased due to the formation of some excessive brominated byproducts. Then, we tried visible light as activator of the racial process. Interestingly, the yield and the selectivity of **2a** increased when a 40 W incandescent light bulb was used at 25 °C for 6 h (Table 1, entry 5) compared to other activation modes.

To make the chemical process green, we designed a bromination process with water as the reaction medium rather than organic solvents. Despite the low solubility of the organic substrates, the yields of **2a** were improved without significant formation of byproducts (Table 1, entries 5 and 6). Furthermore, it was convenient to separate the organic product from the reaction mixtures. In small-scale experiments, a simple extraction with an appropriate organic solvent was efficient to obtain the product. However, in large-scale bromination processes, a clear phase separation occurred, so the product could be obtained by drying the organic phase after separation from the aqueous phase.

Considering the H_2_O_2_ decomposition in the presence of HBr and Br_2_ in the reaction, the effect of the amount of H_2_O_2_ was investigated. Actually, the yields of **2a** increased to 73.1% and 80.4% when 1.5 and 2.0 equiv of H_2_O_2_ were used (Table 1, entries 7 and 8), respectively, in the bromination process. Similarly, when the amount of HBr increased to 1.1 equiv, the yield of **2a** was maximized (Table 1, entry 9). However, a large amount of **2b** was found when excessive HBr (1.5 equiv) was used, which decreased the selectivity of this bromination protocol (Table 1, entry 10).

The effect of reagent addition modes on the bromination yields was also studied. The results showed that gradual addition of H_2_O_2_ (method B) improved the yield of the main product **2a** in contrast to a one-time addition of H_2_O_2_ (method A). This may be due to a significant decrease of H_2_O_2_ decomposition during the slow addition process. In addition, the Br_2_ generated in situ was reduced by stepwise addition of H_2_O_2_, which would improve the selectivity of **2a** by preventing the side reactions.

Next, the bromination of other *para*-xylene derivatives under optimized conditions (see Table 1, entry 11) were investigated to examine the versatility of the protocol. As can be seen in Table 2, *para*-xylene (**3**), 2-chloro-1,4-dimethylbenzene (**5**) and 2-bromo-1,4-dimethylbenzene (**7**) were converted to the corresponding benzyl bromides in high yields with a small amount of dibrominated byproducts. However, in the case of 1-nitro-2,5-dimethylbenzene (**9**), a lower yield of benzyl brominated product was obtained. This could be explained by the deactivating effect of the nitro group [16]. Therefore, a 100 W high pressure mercury lamp (‘solar’ light) was used to increase the formation of bromide radical in the repeated bromination experiment of **9**. On this occasion the yield of the monobrominated product **10a** was high, and this was in agreement with the literature [16].

**Table 2:** Visible-light induced free-radical bromination of substituted *p*-xylenes with H_2_O_2_–HBr.

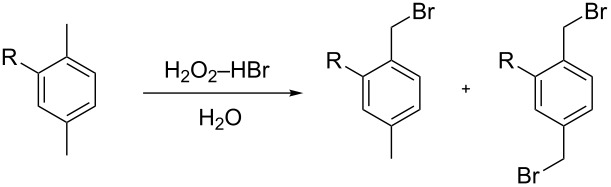		

Substrate	Time(h)	Yield^a^ (%)

**3**: R = H	16	**4a**: 89.2, **4b**: 3.2
**5**: R = Cl	22	**6a**: 85.3, **6b**: 2.5
**7**: R = Br	25	**8a**: 82.7, **8b**: 4.2
**9**: R = NO_2_^b^	60	**10a**: 78.5, **10b**: 2.3

^a^Yields were determined by ^1^H NMR spectroscopy and were based on starting compounds. ^b^The reaction mixture was irradiated with a 100 W high pressure mercury lamp.

**Scheme 1:**
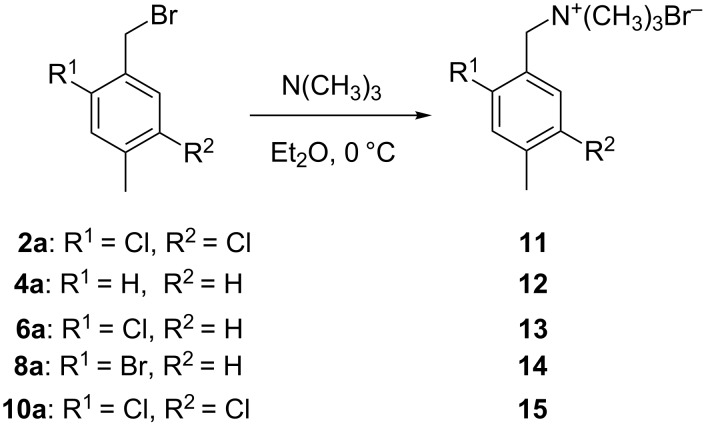
Synthesis of substituted (4-methylbenzyl)trimethylammonium bromides from substituted (4-methylbenzyl)bromides.

Five brominated products were obtained through the above bromination protocol, and were used to synthesize substituted (4-methylbenzyl)trimethylammonium bromides in diethyl ether at 0 °C with quantitative yields [17] (Scheme 1).

Then, we used 2,5-dichloro-(4-methylbenzyl)trimethylammonium bromide (**11**) as starting material to prepare tetrachloro[2.2]paracyclophane in an aqueous sodium hydroxide solution according to Winberg’s method [18–19]. The intermediate 2,5-dichloro-(4-methylbenzyl)trimethylammonium hydroxide was formed and then decomposed in boiling toluene, resulting in a small amount of a dimer product **16** and a quantity of polymer byproduct (Table 3, entry 1). After the reaction, the polymer byproduct was removed by filtration, and the dimer product was obtained by concentrating the filtrate under reduced pressure. Thus, a chromatographic purification was not necessary in the improved dimerization protocol.

**Table 3:** Synthesis of 4,7,12,15-tetrachloro[2.2]paracyclophane **16** from **11**.

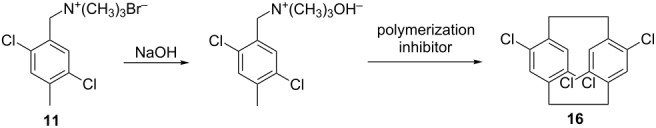		

Entry	Polymerization inhibitor	Yield^a^ (%)

1	–	12
2	phenothiazine	25
3	2-chlorophenothiazine	35

^a^Yields of products were based on compound **11**.

To suppress the polymerization and to improve the yield of the dimer product, we attempted the addition of a polymerization inhibitor. As expected, the addition of 3 mol % phenothiazine significantly improved the yield of **16** to 25% (Table 3, entry 2). The addition of 2-chlorophenothiazine increased the yield to 35% (Table 3, entry 3), which was about three times than that without any inhibitor. In addition, the 35% yield of dimer product was about two times the yield (20%) when the protocol with silver oxide for anion exchange was used [17], and it was comparable to the commercial synthetic protocol with 36.5% yield [20]. Although two isomers from the dimerization reaction could be formed, only the 4,7,12,15-tetrachloro isomer was obtained. The structure of the product was confirmed by ^1^H and ^13^C NMR spectral analysis, and the data matched well with the reported results [17]. Furthermore, the ^1^H NMR spectra of the CH_2_CH_2_ bridge in the paracyclophane structure was consistent with the data reported in the literature, which also identified the 4,7,12,15-tetrachloro isomer [21].

Then, four substituted [2.2]paracyclophanes were synthesized from substituted (4-methylbenzyl)trimethylammonium bromides in aqueous sodium hydroxide solution in the presence of a polymerization inhibitor (Table 4). It was found that the yields of dimer products were improved dramatically compared to the results obtained with silver oxide used for anion exchange reported by Chow [17]. We speculated that the replacement of silver oxide by aqueous sodium hydroxide solution might promote the formation of substituted (4-methylbenzyl)trimethylammonium hydroxide, but we are unable to provide any conclusive evidence at presence. For the dimerization of **12**, the [2.2]paracyclophane (**17**) was obtained in 33% yield, and its structure was confirmed by NMR spectroscopy and elemental analysis. Similarly, dimerization of **13**, **14**, and **15** resulted in regiospecific 4,16-disubstituted [2.2]paracyclophanes **18**, **19**, and **20**, respectively, in about 35% yield (Table 4, entries 2, 3 and 4). The structures of the synthesized 4,16-disubstituted [2.2]paracyclophanes were also consistent with their NMR spectral data.

**Table 4:** Synthesis of substituted [2.2]paracyclophanes from substituted (4-methylbenzyl)trimethylammonium bromides.

Entry	Starting material	Product	Yield^a^ (%)

1	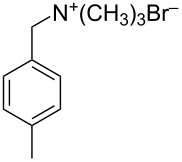 **12**	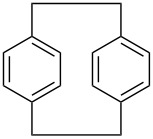 **17**	33 (23)
2	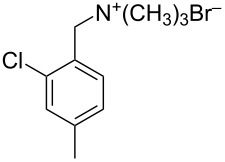 **13**	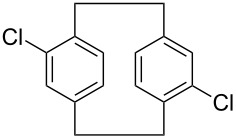 **18**	36 (24)
3	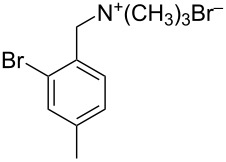 **14**	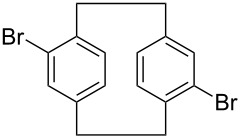 **19**	33 (19)
4	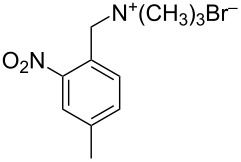 **15**	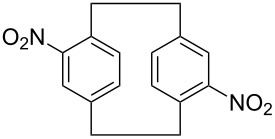 **20**	32 (18)

^a^In the presence of 2-chlorophenothiazine. The numbers in parenthesis are the yields in the presence of phenothiazine.

## Conclusion

A convenient protocol was reported to synthesize 4,7,12,15-tetrachloro[2.2]paracyclophane. In the first bromination step, 1-(bromomethyl)-2,5-dichloro-4-methylbenzene was synthesized with high yield and selectivity from 2,5-dichloro-*p*-xylene by using a H_2_O_2_–HBr couple in water. The use of H_2_O_2_–HBr as a bromide source made this procedure organic-waste-free, organic-solvent-free and an appropriate replacement of the existing bromination methods. In the Winberg elimination–dimerization step, 35% yield of 4,7,12,15-tetrachloro[2.2]paracyclophane was obtained from 2,5-dichloro-(4-methylbenzyl)trimethylammonium bromide and aqueous sodium hydroxide solution in the presence of a polymerization inhibitor, which was about two folds than that used silver oxide as anion exchange. Moreover, four substituted [2.2]paracyclophanes were prepared in this convenient way.

## Experimental

### General

2,5-Dichloro-*p*-xylene, *para*-xylene, 2-chloro-1,4-dimethylbenzene, 2-bromo-1,4-dimethylbenzene and 1-nitro-2,5-dimethylbenzene were purchased from commercial suppliers. All chemicals were used as received without further purification. ^1^H NMR spectra were recorded in CDCl_3_ using an AVANCE III 400WB spectrometer. IR spectra were recorded on a Nicolet AVATAR 5700 FTIR spectrophotometer in the range of 4000–400 cm^−1^ using KBr pellets. Melting points were determined using a Beijing TaiKe X-4 melting point apparatus and were uncorrected. Mass spectra were obtained using an Agilent 1260-6224 spectrometer with electron impact ionization (EI, 70 eV). Elemental analyses were recorded on an Elementar vario MICRO cube.

### Typical reaction procedure for visible-light induced bromination with the H_2_O_2_–HBr system

Analogous as described in [16], substituted *p*-xylene (1.0 mmol) was added to 2.0 mL solution (CCl_4_ or water) of 2.0 mmol of H_2_O_2_ (0.23 g, 30% H_2_O_2_ aqueous) and 1.1 mmol of HBr (0.22 g, 30% HBr aqueous). The mixture was stirred at 300 rpm at appropriate temperature under irradiation from a 40 W incandescent light bulb. At the end of the bromination reaction (6–20 h), the mixture was transferred into a separating funnel and 4 mL of 0.005 M NaHSO_3_ was added. The crude product was extracted using 3 × 5 mL CH_2_Cl_2_ and the combined organic phase was dried over MgSO_4_. Then the solvent was evaporated under reduced pressure and the crude mixture was analyzed by ^1^H NMR spectroscopy. Lastly the products were separated by column chromatography (SiO_2_, hexane/EtOAc) and identified by comparison with literature data.

**2a**: colorless oil. ^1^H NMR (CDCl_3_) δ 2.24 (s, 3H, ArCH_3_), 5.12 (s, 2H, ArCH_2_), 7.29 (s, 1H, ArH), 7.33 (s, 1H, ArH); EIMS *m*/*z*: 254, 175, 173, 102.

**4a**: colorless oil. ^1^H NMR (CDCl_3_) δ 2.19 (s, 3H, ArCH_3_), 4.66 (s, 2H, ArCH_2_), 7.07–7.11 (m, 2H, ArH), 7.25–7.31 (m, 1H, ArH); anal. calcd for C_8_H_9_Br (185.06): C, 51.92; H, 4.90; found: C, 51.81; H, 4.96.

**6a**: colorless oil. ^1^H NMR (CDCl_3_) δ 2.31 (s, 3H, ArCH_3_), 4.95 (s, 2H, ArCH_2_), 6.96–6.98 (m, 1H, ArH), 6.99–7.24 (m, 1H, ArH), 7.26–7.37 (m, 1H, ArH); anal. calcd for C_8_H_8_BrCl (219.51): C, 43.77; H, 3.67; Cl, 16.15; found: C, 43.68; H, 3.72; Cl, 16.06.

**8a**: mp 53–55 °C; ^1^H NMR (CDCl_3_) δ 2.31 (s, 3H, ArCH_3_), 4.93 (s, 2H, ArCH_2_), 7.02–7.54 (m, 3H, ArH); anal. calcd for C_8_H_8_BrCl (219.51): C, 43.77; H, 3.67; Cl, 16.15; found: C, 43.68; H, 3.72; Cl, 16.06.

**10a**: mp 72–74 °C; ^1^H NMR (CDCl_3_) δ 2.41 (s, 3H, ArCH_3_), 4.95 (s, 2H, ArCH_2_), 6.96–7.37 (m, 3H, ArH); anal. calcd for C_8_H_8_BrNO_2_ (230.06): C, 41.77; H, 3.51; N, 6.09; found: C, 41.68; H, 3.57; N, 6.12.

### Typical reaction procedure for the preparation of substituted (4-methylbenzyl)trimethylammonium bromides

Substituted 4-methylbenzyl bromide (5.0 mmol) was added to 50.0 mL Et_2_O solution in a 100 mL three-necked flask. The mixture was cooled at 0 °C and was stirred at 300 rpm. Me_3_N was generated by heating an aqueous Me_3_N solution (40% w/w, 15 mL) and passed into the flask for 4 h. The product was precipitated as a white solid. Then the mixture was stirred at room temperature overnight and the quaternary ammonium salt was obtained on a Büchner funnel and dried in a vacuum oven at 80 °C for 24 h.

**11**: highly hygroscopic solid. IR (KBr) ν/cm^−1^: 3004, 1635, 1617, 1477, 1375, 1190, 980.

**12**: highly hygroscopic solid. IR (KBr) v/cm^−1^: 2989, 1521, 1483, 1382, 1125, 910, 805, 722.

**13**: highly hygroscopic solid. IR (KBr) v/cm^−1^: 2968, 2935, 1632, 1452, 1371, 1154, 725, 672.

**14**: highly hygroscopic solid. IR (KBr) v/cm^−1^: 3009, 2946, 1642, 1458, 1381, 1205, 653.

**15**: highly hygroscopic solid. IR (KBr) v/cm^−1^: 2979, 1621, 1550, 1508, 1472, 1376, 1345, 1135, 663.

### Typical reaction procedure for the synthesis of substituted tetrachloro[2.2]paracyclophanes

In a 100 mL three-necked flask equipped with a stirrer and a Dean–Stark water separator attached to a reflux condenser was placed 15 mL aqueous sodium hydroxide solution (40% w/w) and 45 mL toluene. With vigorous stirring, a solution of benzyltrimethylammonium bromides (50 mmol), dissolved in 5 mL water, was added dropwise in 30 min. The inhibitor (0.15 mmol) was then added to the solution and the mixture was heated under reflux for 4 h. After all water had been separated, a pale yellow solid polymer began to precipitate. When the evolution of Me_3_N was finished, the reaction system was heated and stirred for another 1 h. The mixture was cooled and the solid was filtrated and washed with toluene (5 mL × 3). The filtrates were combined and evaporated under vacuum to give a solid product which was further washed with hexane (5 mL × 3).

**16**: white solid, mp >280 °C (dec); ^1^H NMR (CDCl_3_) δ 2.91 (m, 2H, ArCH_2_), 3.26 (m, 2H, ArCH_2_), 6.95 (s, 2H, ArH); ^13^C NMR (CDCl_3_) δ 30.8, 77.0, 131.8, 133.9, 138.6; anal. calcd for C_16_H_12_Cl_4_ (346.07): C, 55.53; H, 3.50; Cl, 40.97; found: C, 55.47; H, 3.62; Cl, 40.89.

**17**: white solid, mp 281–283 °C; ^1^H NMR (CDCl_3_) δ 3.09 (s, 8H, ArCH_2_), 6.50 (s, 8H, ArH); anal. calcd for C_16_H_16_ (208.30): C, 92.26; H, 7.74; found: C, 92.15; H, 7.82.

**18**: white solid, mp 163–165 °C; ^1^H NMR (CDCl_3_) δ 2.85–2.97 (m, 4H, ArCH_2_), 3.03–3.37 (m, 4H, ArCH_2_), 6.92–7.54 (m, 6H, ArH). anal. calcd for C_16_H_14_Cl_2_ (277.19): C, 69.33; H, 5.09; Cl, 25.58; found: C, 69.27; H, 5.05; Cl, 25.65.

**19**: white solid, mp 238–240 °C; ^1^H NMR (CDCl_3_) δ 2.86–3.12 (m, 4H, ArCH_2_), 3.15–3.34 (4H, m, ArCH_2_), 6.43–7.15 (m, 6H, ArH); anal. calcd for C_16_H_14_Br_2_ (366.10): C, 52.49; H, 3.85; found: C, 52.38; H, 3.82.

**20:** ^1^H NMR (CDCl_3_) δ 2.81–3.07 (m, 4H, ArCH_2_), 3.27–3.35 (m, 4H, ArCH_2_), 7.25–8.23 (m, 6H, ArH); anal. calcd for C_16_H_14_N_2_O_4_ (298.30): C, 64.42; H, 4.73; N, 9.39; found: C, 64.32; H, 4.75; N, 9.45.

## Supporting Information

File 1 Copies of MS, ^1^H and ^13^C NMR spectra of the synthesized compounds.
