# Supplementary material for: A new protocol for the synthesis of 4,7,12,15-tetrachloro[2.2]paracyclophane
Source: Beilstein J Org Chem. 2016 Nov 17;12:2443–9. doi: 10.3762/bjoc.12.237 (PMC5238561; doi:10.3762/bjoc.12.237)

Supporting Information

for

A new protocol for the synthesis of 4,7,12,15-tetrachloro[2.2]paracyclophane

Donghui Pan, Yanbin Wang and Guomin Xiao*

Address: School of Chemistry and Chemical Engineering, Southeast University, 2 Dongnan Daxue Road, Nanjing, Jiangsu, 211189, P. R. China

Email: Guomin Xiao* - xiaogm426@gmail.com

* Corresponding author

Copies of MS, ^{1}H and ^{13}C NMR spectra of the synthesized compounds

1. ^{1}H NMR spectra of **2a–10a**……………………………………………..S2–S4

2. MS spectrum of **2a**……………………………………………………………...S5

3. ^{1}H NMR and ^{13}C NMR spectra of **16** …………………………………………S6

4. ^{1}H NMR spectra of **17–20**……………………………………………………S7

^{1}H NMR spectrum of 1-(bromomethyl)-2,5-dichloro-4-methylbenzene (**2a**)

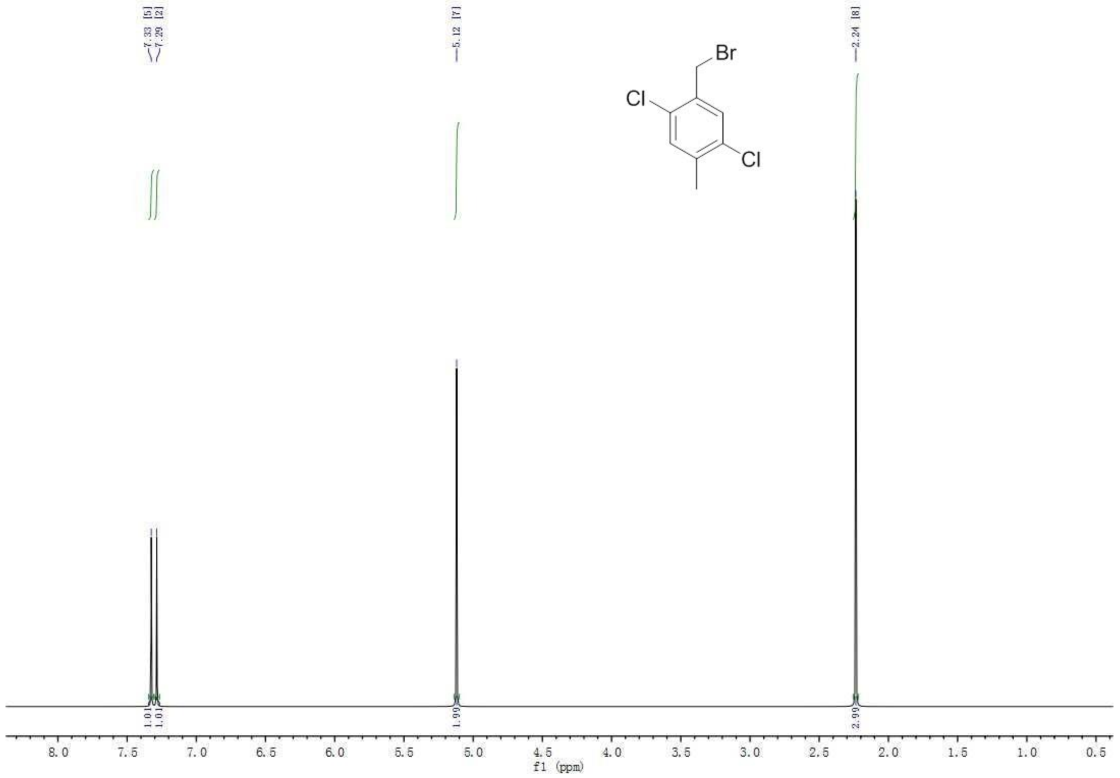

^{1}H NMR spectrum of 1-(bromomethyl)- 4-methylbenzene (**4a**)

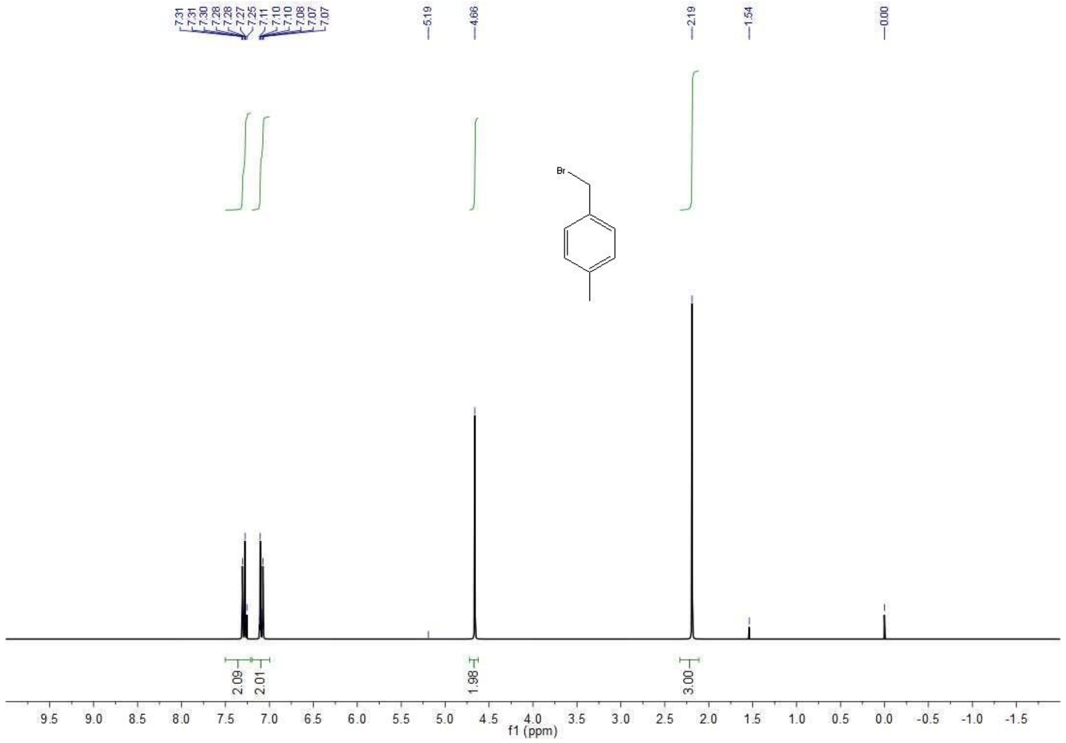

^{1}H NMR spectrum of 1-(bromomethyl)-2-chloro-4-methylbenzene (**6a**)

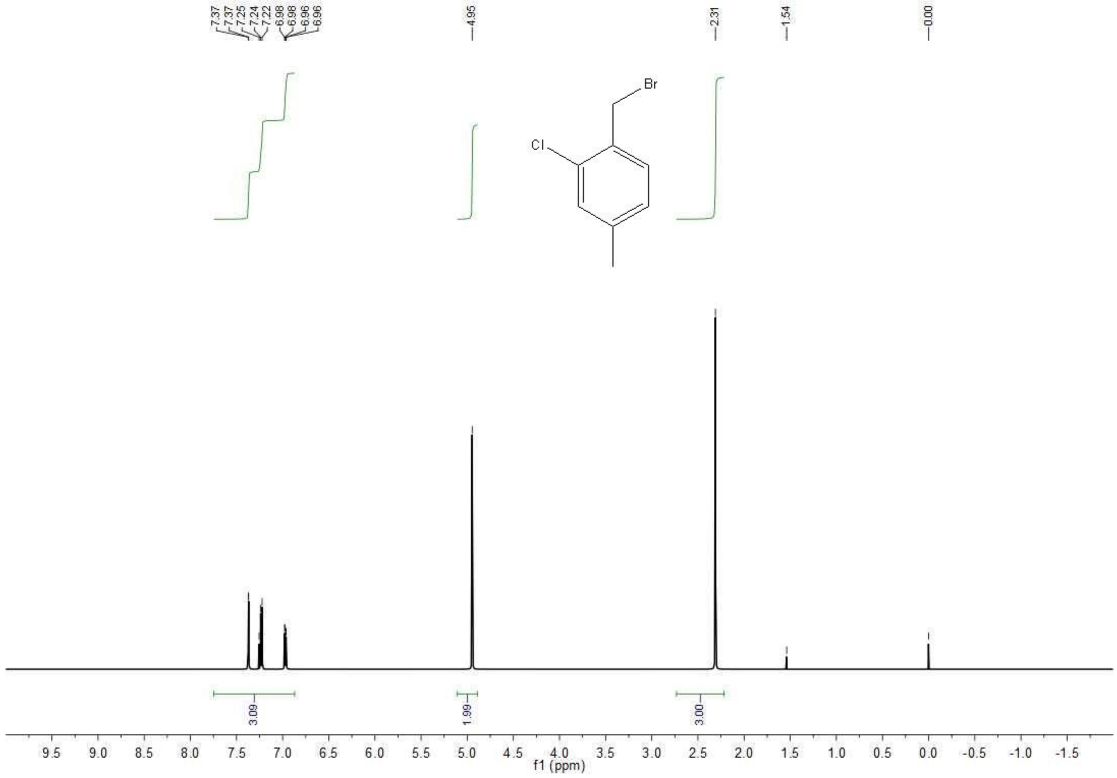

^{1}H NMR spectrum of 1-(bromomethyl)-2-bromo-4-methylbenzene (**8a**)

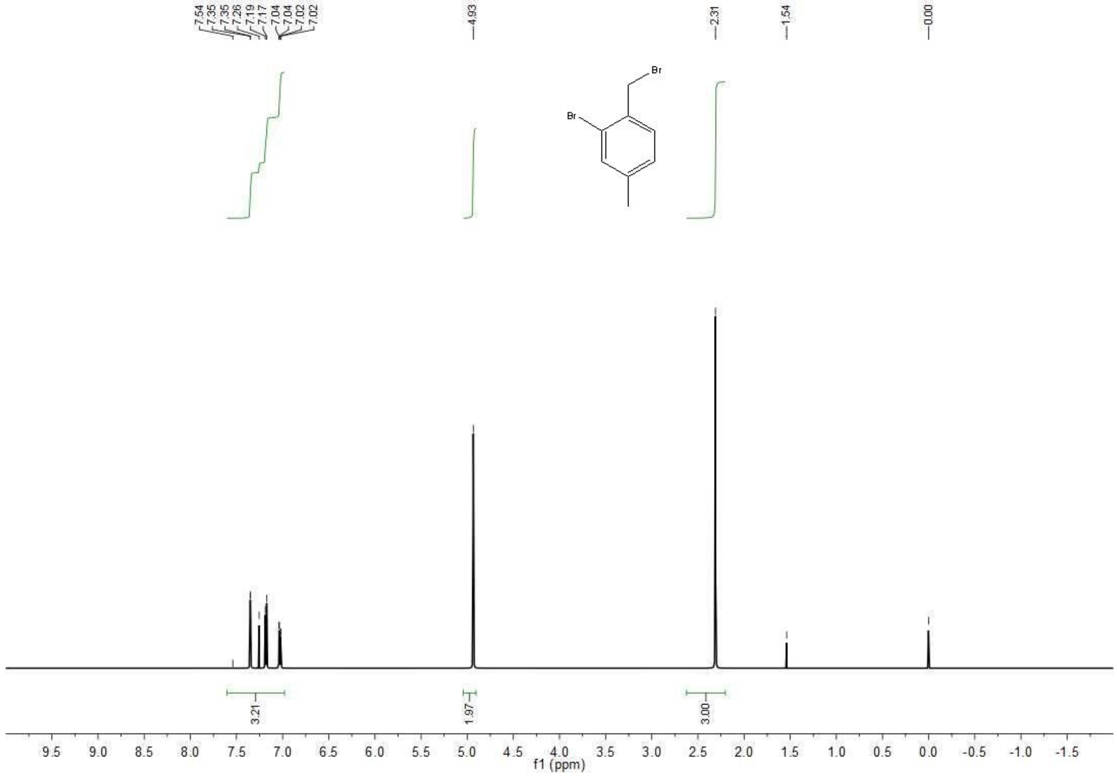

^{1}H NMR spectrum of 1-(bromomethyl)-2-nitro-4-methylbenzene (**10a**)

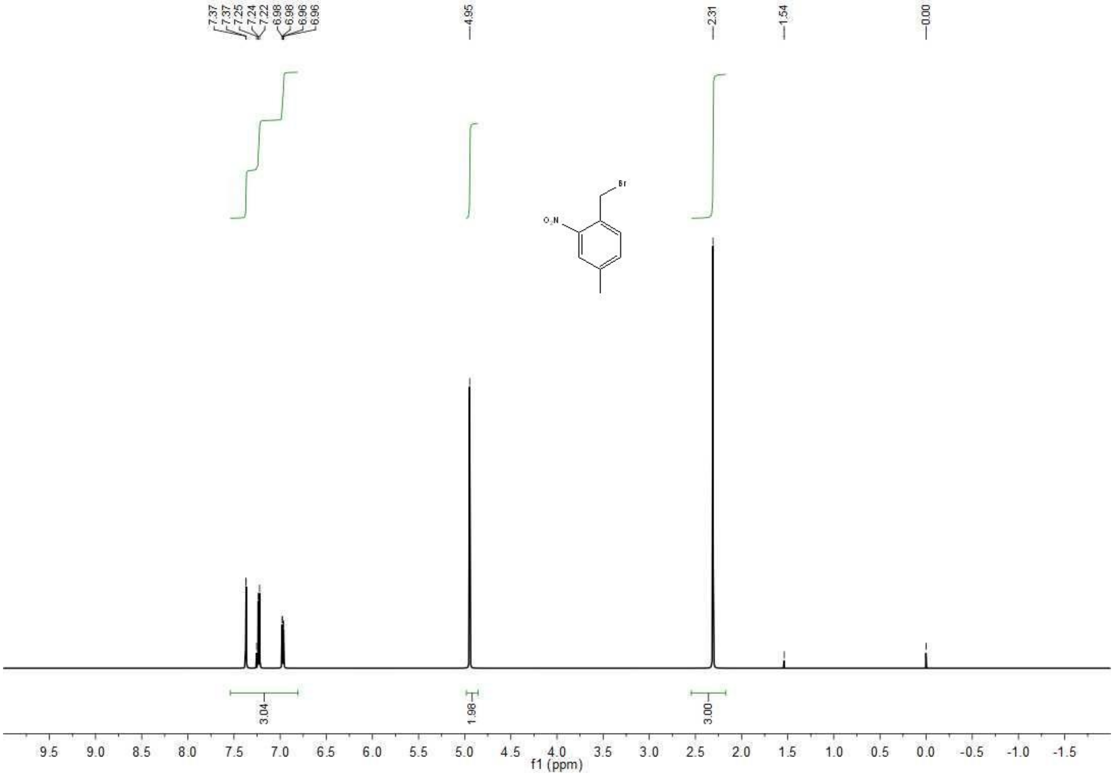

Mass spectrum of 1-(bromomethyl)-2,5-dichloro-4-methylbenzene (**2a**)

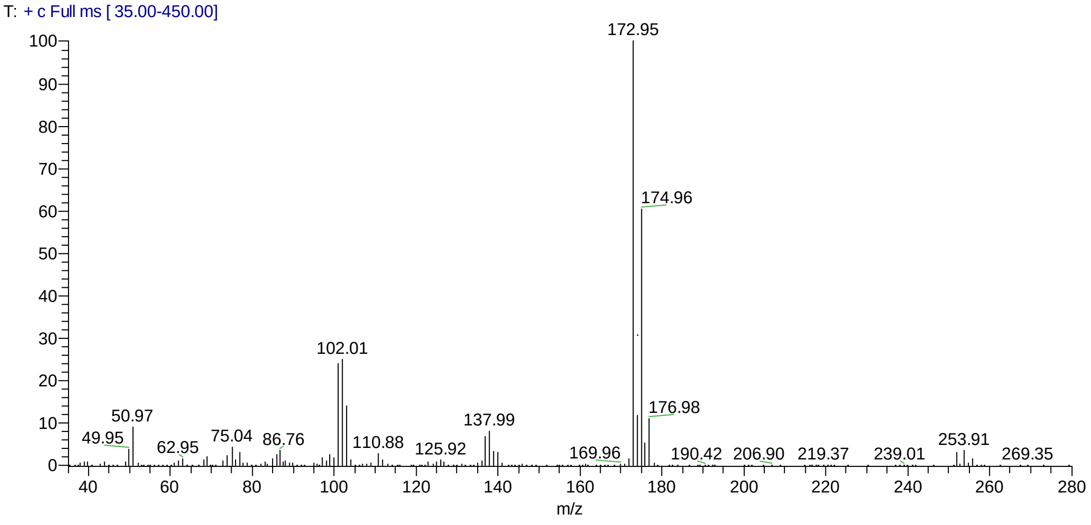

^{1}H NMR spectrum of 4,7,12,15-tetrachloro[2.2]paracyclophane (**16**)

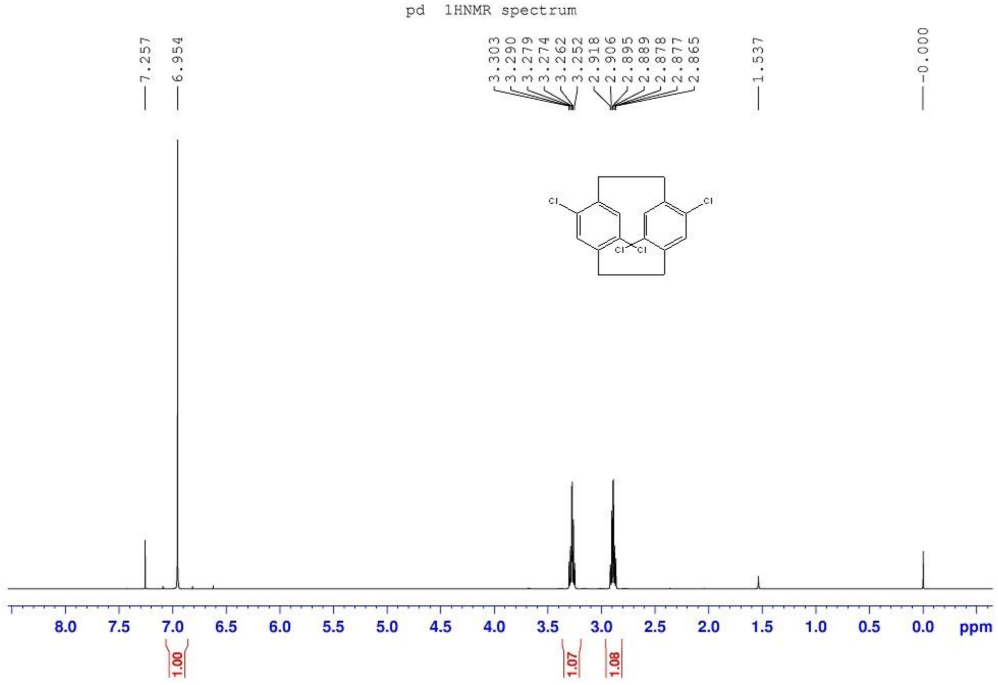

^{13}C NMR spectrum of 4,7,12,15-tetrachloro[2.2]paracyclophane (**16**)

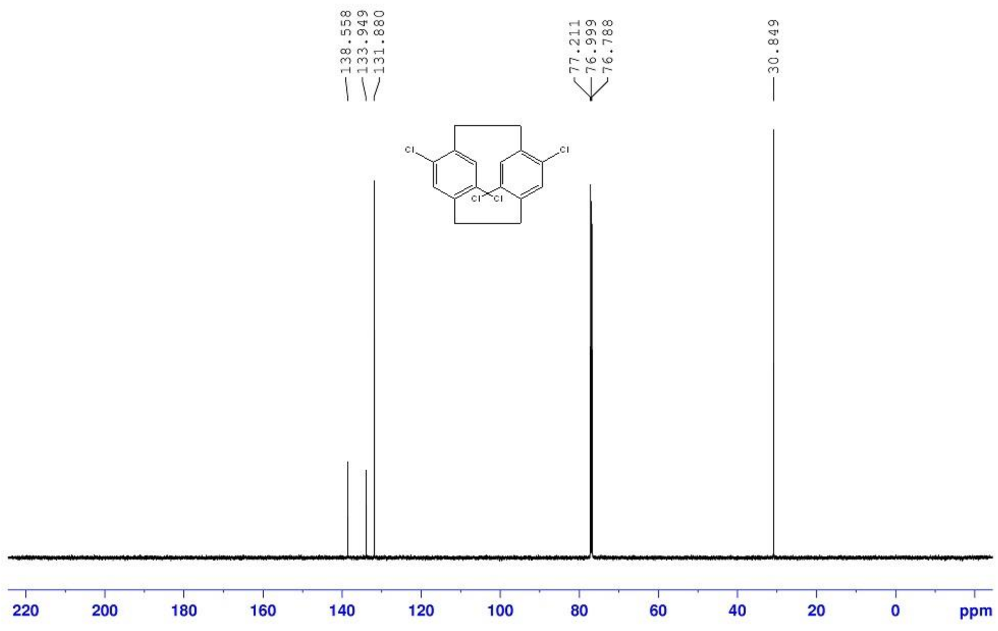

^{1}H NMR spectrum of [2.2]paracyclophane (**17**)

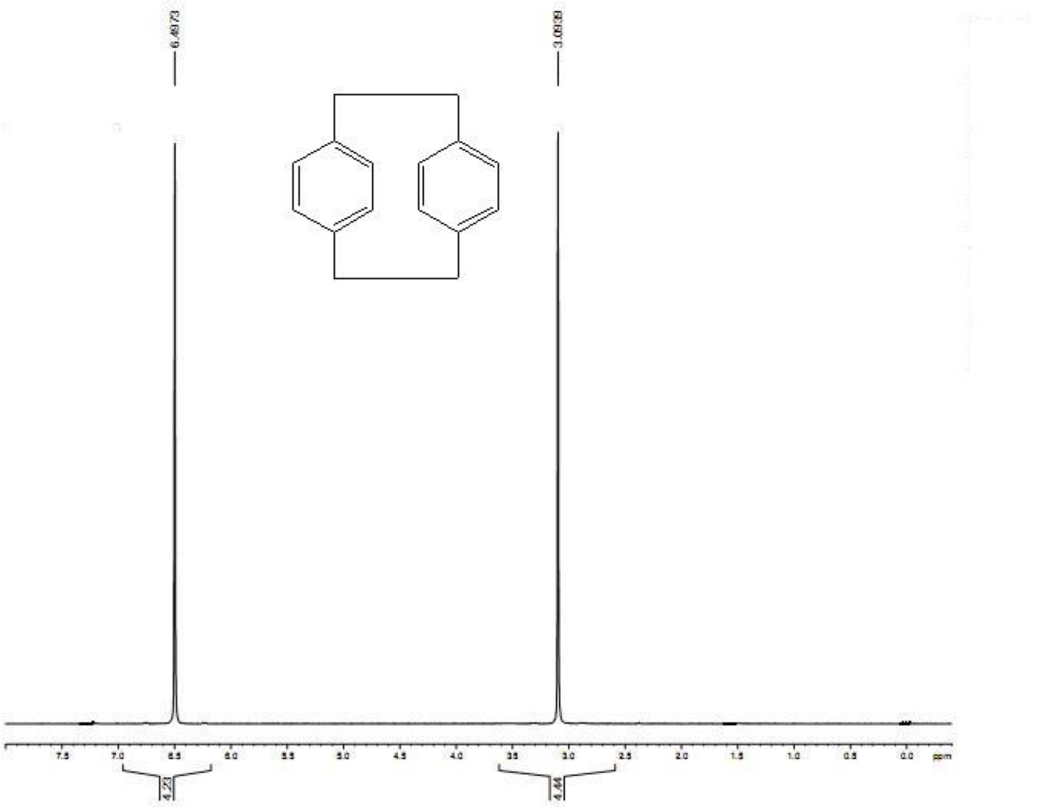

^{1}H NMR spectrum of 4,16-dichloro[2.2]paracyclophane (**18**)

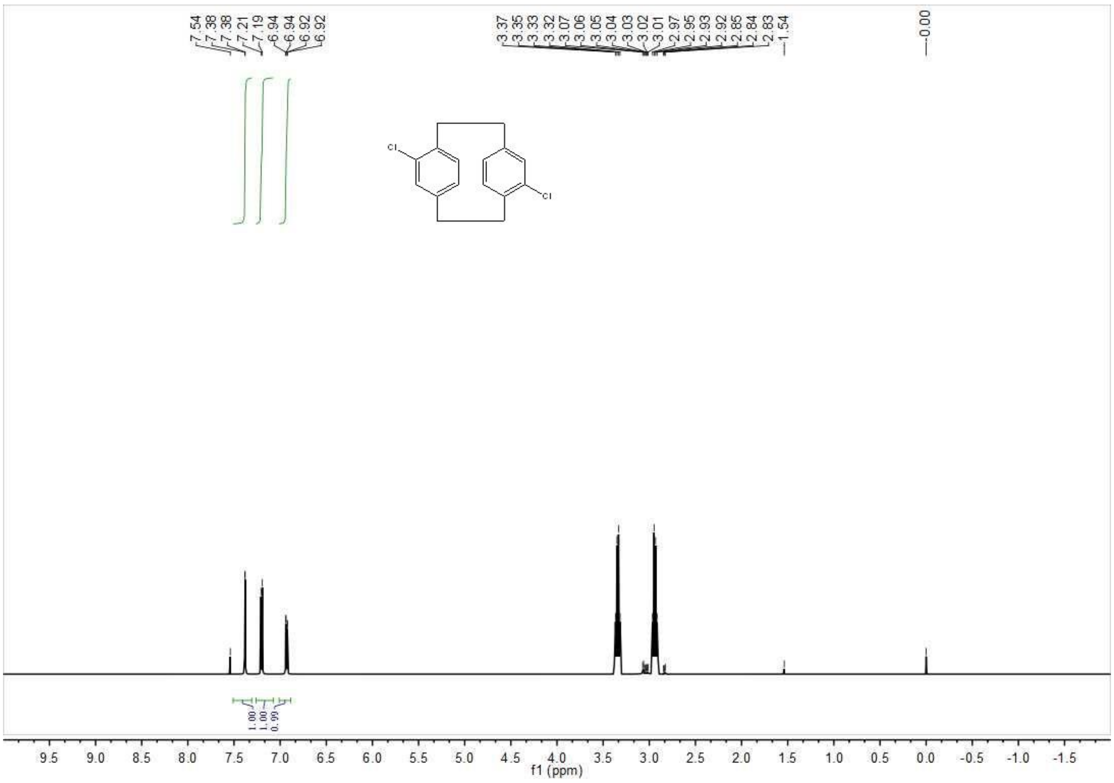

^{1}H NMR spectrum of 4,16-dibromo[2.2]paracyclophane (**19**)

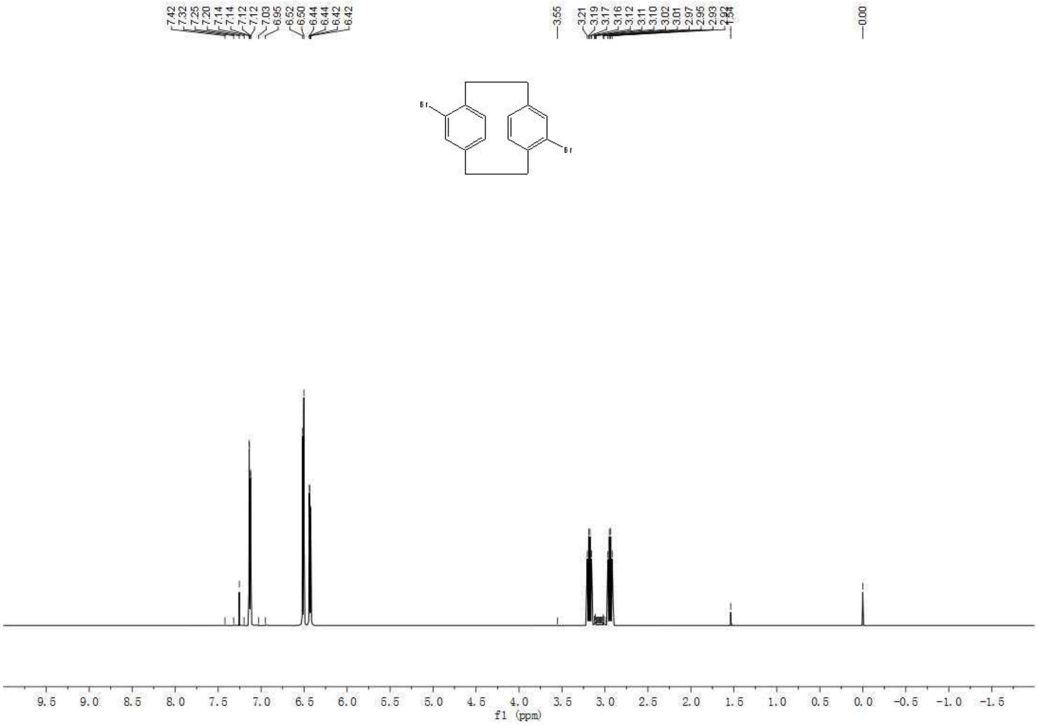

^{1}H NMR spectrum of 4,16-dinitro[2.2]paracyclophane (**20**)

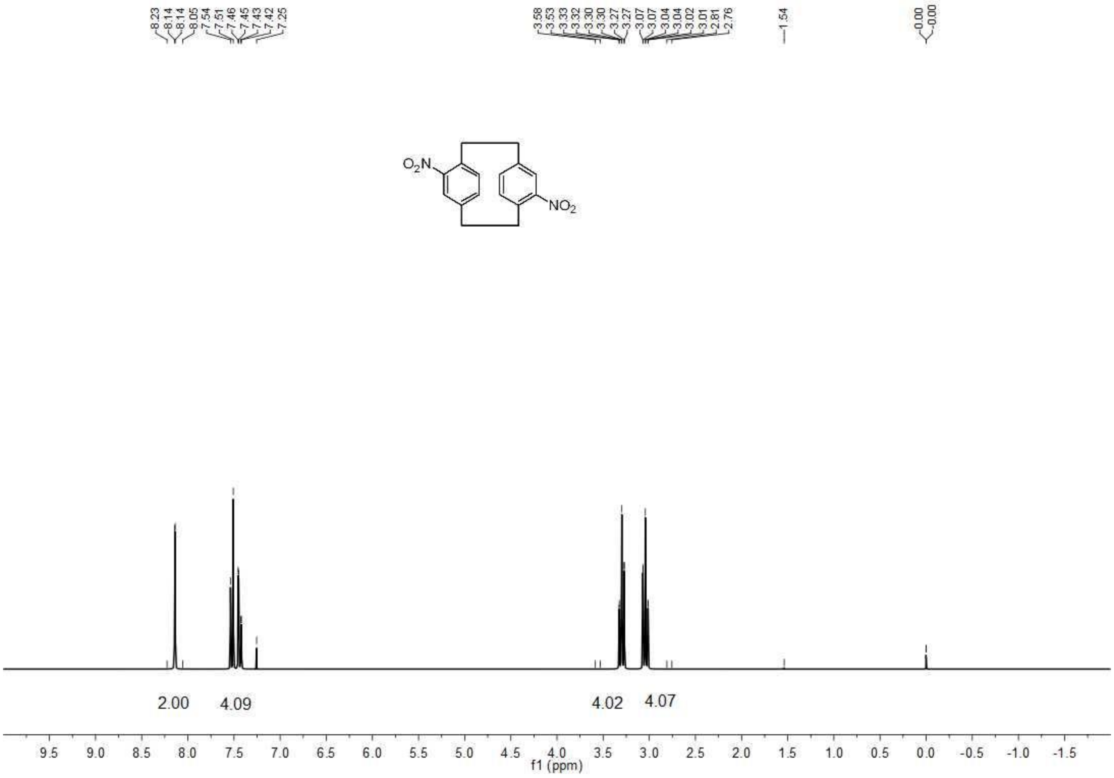

Supplement: File 1 — Copies of MS, 1H and 13C NMR spectra of the synthesized compounds. [file Beilstein_J_Org_Chem-12-2443-s001.pdf]
